# Supplementary material for: P-TRAP: a Panicle Trait Phenotyping tool
Source: BMC Plant Biol. 2013 Aug 29;13:122. doi: 10.1186/1471-2229-13-122 (PMC3848748; doi:10.1186/1471-2229-13-122)
Supplement: Additional file 7 — P-TRAP source code. The Netbeans project that contains the source code of the application. [file 1471-2229-13-122-S7.zip › javadoc.zip!/javadoc/org-biolag-particleprocessor/org/biolag/particleprocessor/ConcavePoint.html]

ConcavePoint (ParticleProcessor)


JavaScript is disabled on your browser.


- Package
- Class
- Tree
- Deprecated
- Index
- Help

*org.biolag.particleprocessor 1.0*

- Prev Class
- Next Class

- Frames
- No Frames

- All Classes

- Summary:
- Nested |
- Field |
- Constr |
- Method

- Detail:
- Field |
- Constr |
- Method


org.biolag.particleprocessor

## Class ConcavePoint

- java.lang.Object
- - java.awt.geom.Point2D
  - - java.awt.Point
    - - org.biolag.particleprocessor.ConcavePoint

- All Implemented Interfaces:
  :   java.io.Serializable, java.lang.Cloneable

  ---

    

  ```
  public class ConcavePoint
  extends java.awt.Point
  ```

  Author:
  :   faroq

  See Also:
  :   Serialized Form

- - ### Nested Class Summary

    - ### Nested classes/interfaces inherited from class java.awt.geom.Point2D

      `java.awt.geom.Point2D.Double, java.awt.geom.Point2D.Float`
  - ### Field Summary

    - ### Fields inherited from class java.awt.Point

      `x, y`
  - ### Constructor Summary

    Constructors

    Constructor and Description

    `ConcavePoint(double concavness,
    int index,
    int i,
    int i1)`

    `ConcavePoint(double concavness,
    int index,
    java.awt.Point point)`
  - ### Method Summary

    Methods

    | Modifier and Type | Method and Description |
    | --- | --- |
    | `double` | `getConcavness()` |
    | `int` | `getIndex()` |
    | `void` | `setConcavness(double concavness)` |
    | `void` | `setIndex(int index)` |

    - ### Methods inherited from class java.awt.Point

      `equals, getLocation, getX, getY, move, setLocation, setLocation, setLocation, toString, translate`
    - ### Methods inherited from class java.awt.geom.Point2D

      `clone, distance, distance, distance, distanceSq, distanceSq, distanceSq, hashCode, setLocation`
    - ### Methods inherited from class java.lang.Object

      `finalize, getClass, notify, notifyAll, wait, wait, wait`

- - ### Constructor Detail


    - #### ConcavePoint

      ```
      public ConcavePoint(double concavness,
                  int index,
                  int i,
                  int i1)
      ```


    - #### ConcavePoint

      ```
      public ConcavePoint(double concavness,
                  int index,
                  java.awt.Point point)
      ```
  - ### Method Detail


    - #### getConcavness

      ```
      public double getConcavness()
      ```


    - #### setConcavness

      ```
      public void setConcavness(double concavness)
      ```


    - #### getIndex

      ```
      public int getIndex()
      ```


    - #### setIndex

      ```
      public void setIndex(int index)
      ```


- Package
- Class
- Tree
- Deprecated
- Index
- Help

*org.biolag.particleprocessor 1.0*

- Prev Class
- Next Class

- Frames
- No Frames

- All Classes

- Summary:
- Nested |
- Field |
- Constr |
- Method

- Detail:
- Field |
- Constr |
- Method
